# Internet addiction: coping styles, expectancies, and treatment implications

**DOI:** 10.3389/fpsyg.2014.01256

**Published:** 2014-11-11

**Authors:** Matthias Brand, Christian Laier, Kimberly S. Young

**Affiliations:** ^1^Department of General Psychology: Cognition, University of Duisburg-EssenDuisburg, Germany; ^2^Erwin L. Hahn Institute for Magnetic Resonance ImagingEssen, Germany; ^3^Center for Internet Addiction, Russell J. Jandoli School of Journalism and Mass Communication, St. Bonaventure UniversityOlean, NY, USA

**Keywords:** Internet addiction, personality, psychopathology, coping, cognitive-behavioral therapy

## Abstract

Internet addiction (IA) has become a serious mental health condition in many countries. To better understand the clinical implications of IA, this study tested statistically a new theoretical model illustrating underlying cognitive mechanisms contributing to development and maintenance of the disorder. The model differentiates between a generalized Internet addiction (GIA) and specific forms. This study tested the model on GIA on a population of general Internet users. The findings from 1019 users show that the hypothesized structural equation model explained 63.5% of the variance of GIA symptoms, as measured by the short version of the Internet Addiction Test. Using psychological and personality testing, the results show that a person’s specific cognitions (poor coping and cognitive expectations) increased the risk for GIA. These two factors mediated the symptoms of GIA if other risk factors were present such as depression, social anxiety, low self-esteem, low self-efficacy, and high stress vulnerability to name a few areas that were measured in the study. The model shows that individuals with high coping skills and no expectancies that the Internet can be used to increase positive or reduce negative mood are less likely to engage in problematic Internet use, even when other personality or psychological vulnerabilities are present. The implications for treatment include a clear cognitive component to the development of GIA and the need to assess a patient’s coping style and cognitions and improve faulty thinking to reduce symptoms and engage in recovery.

## INTRODUCTION

A problematic use of the Internet has been identified in a number of studies and shows that persistent negative consequences such as job loss, academic failure, and divorce resulted from excessive Internet use (for reviews see Griffiths, 2000a,b; Chou et al., 2005; Widyanto and Griffiths, 2006; Byun et al., 2009; Weinstein and Lejoyeux, 2010; Lortie and Guitton, 2013). The clinical relevance of this phenomenon gains in importance against the background of high estimated prevalence rates ranging from 1.5 to 8.2% (Weinstein and Lejoyeux, 2010) or even up to 26.7%, depending on the scales used and criteria applied (Kuss et al., 2014).

Although the first description of this clinical issue is almost 20 years ago (Young, 1996), the classification is still discussed controversially and consequently several terms are used in the scientific literature, ranging from “compulsive Internet use” (Meerkerk et al., 2006, 2009, 2010), “Internet related problems” (Widyanto et al., 2008), “problematic Internet use” (Caplan, 2002), “pathological Internet use” (Davis, 2001) to “Internet related addictive behavior” (Brenner, 1997), to mention just a few. In the last 10 years, however, most researchers in this field have used the term “Internet addiction” or “Internet addiction disorder” (e.g., Johansson and Götestam, 2004; Block, 2008; Byun et al., 2009; Dong et al., 2010, 2011, 2013; Kim et al., 2011; Purty et al., 2011; Young, 2011b, 2013; Young et al., 2011; Zhou et al., 2011; Cash et al., 2012; Hou et al., 2012; Hong et al., 2013a,b; Kardefelt-Winther, 2014; Pontes et al., 2014; Tonioni et al., 2014). We also prefer the term “Internet addiction (IA),” because recent articles (see discussion in Brand et al., 2014) highlight the parallels between an overuse of the Internet and other addictive behaviors (e.g., Grant et al., 2013) and also substance dependency (see also Young, 2004; Griffiths, 2005; Meerkerk et al., 2009). It has been argued that mechanisms related to the development and maintenance of substance dependency are transferrable to an addictive use of Internet applications (and also other behavioral addictions), for example the incentive sensitization theory of addiction and related concepts (e.g., Robinson and Berridge, 2000, 2001, 2008; Berridge et al., 2009). This fits also nicely with the component model on addictive behaviors (Griffiths, 2005).

Many studies have been conducted on psychological correlates of IA, but this has been done – at least in most cases – without differentiating between a generalized Internet addiction (GIA) and a specific Internet addiction (SIA; Morahan-Martin and Schumacher, 2000; Leung, 2004; Ebeling-Witte et al., 2007; Lu, 2008; Kim and Davis, 2009; Billieux and Van der Linden, 2012), although psychological mechanisms might be different, also for distinct age groups or applications used (Lopez-Fernandez et al., 2014). Our study examines the mediating effects of coping styles and cognitive expectations for Internet use in the development and maintenance of GIA in order to contribute to a better understanding of underlying mechanisms and potential implications for diagnostic and treatment.

On a theoretical level, it was already postulated that IA has to be differentiated regarding the generalized Internet use (Griffiths and Wood, 2000) versus specific types of IA such as cybersex, online relations, net compulsions (e.g., gambling, shopping), information search, and online gaming for developing an addiction to the Internet (e.g., Young et al., 1999; Meerkerk et al., 2006; Block, 2008; Brand et al., 2011). However, only one subtype, Internet Gaming Disorder, has been included in the appendix of the DSM-5 (APA, 2013). Most studies either assessed IA as a unified construct or only assessed one specific subtype (in most cases Internet gaming). In his cognitive-behavioral model, Davis (2001) also differentiated between a generalized pathological Internet use (GIA) and a specific pathological Internet use (SIA). GIA was described as a multidimensional overuse of the Internet, frequently accompanied by time waste and non-directed use of the Internet. Social aspects of the Internet (e.g., social communication via social networking sites) are particularly used (see also discussion in Lortie and Guitton, 2013), which is supposed to be linked to a lack of social support oﬄine and social deficits experienced by an individual in non-virtual situations. In addition, it has been argued that subjects may use several different Internet applications excessively without having one certain favorite, for example playing games, watching pornography, surfing on information and/or shopping sites, posting selfies, watching videos on video platforms, reading blogs of others, and so on. In this case, one may argue that the individual is addicted to the Internet and not addicted on the Internet (but see also discussion in Starcevic, 2013). Davis argues that one main difference between GIA and SIA is that individuals who suffer from GIA would not have developed a similar problematic behavior without the Internet, whereas individuals suffering from SIA would have developed similar problematic behavior within another setting. In both forms of addictive use of the Internet, GIA and SIA, dysfunctional cognitions about the self and about the world are suggested to play a fundamental role (Caplan, 2002, 2005).

Research addressing GIA demonstrated that subjective complaints in everyday life resulting from Internet use are correlated with diverse personality characteristics. Indeed, it was shown that GIA is linked to psychopathological comorbidities, such as affective or anxiety disorders (Whang et al., 2003; Yang et al., 2005; Weinstein and Lejoyeux, 2010) as well as to the personality traits shyness, neuroticism, stress vulnerability, tendencies to procrastinate, and low self-esteem (Niemz et al., 2005; Ebeling-Witte et al., 2007; Hardie and Tee, 2007; Thatcher et al., 2008; Kim and Davis, 2009). Also, factors of social context, e.g., lack of social support or social isolation (Morahan-Martin and Schumacher, 2003; Caplan, 2007) and even loneliness in the educational setting in adolescents (Pontes et al., 2014), seem to be related to GIA. Moreover, it has been argued that using the Internet as a tool for coping with problematic or stressful life events contributes to the development of GIA (Whang et al., 2003; Tang et al., 2014). Persons with IA show also high tendency toward impulsive coping strategy (Tonioni et al., 2014). Some authors even conceptualize IA as a type of coping with everyday life or daily hassles (Kardefelt-Winther, 2014). There are still only some first studies, which explicitly compared predictors of different types of SIA. Pawlikowski et al. (2014) reported that shyness and life satisfaction are related to an addictive use of Internet games, but not to a pathological use of cybersex or the use of both games and cybersex.

Based on previous research, in particular on the arguments by Davis (2001), and also considering current literature on neuropsychological and neuroimaging findings in subjects who are addicted on the Internet, we have recently published a theoretical model on the development and maintenance of GIA and SIA (Brand et al., 2014). Some aspects included in the model have already been mentioned in the context of the use of social networking sites, for example the expectancy of positive outcomes (Turel and Serenko, 2012). It has also been shown that an excessive or addictive use of online auctions is correlated with changes in individuals’ beliefs about the technique and this determinates future use and use intentions (Turel et al., 2011). This is in line with our theoretical model on GIA, in which we assume that beliefs or expectancies about what the Internet can do for a person influence the behavior, i.e., the Internet use, which in turn also influences future expectancies. However, in our model we have focused on the mediating role of expectancies and coping strategies in developing and maintaining a GIA and specific types of SIA.

For the development and maintenance of GIA, we argue that the user has certain needs and goals which can be achieved by using certain Internet applications. Based on prior research, we incorporated several of those findings to develop a comprehensive model to tie these elements together. Initially, a person’s core characteristics are associated with IA and include psychopathological aspects, personality aspects, and social cognitions. In the first section, we included psychopathological symptoms, in particular depression and social anxiety (e.g., Whang et al., 2003; Yang et al., 2005), dysfunctional personality facets, such as low self-efficacy, shyness, stress vulnerability, and procrastination tendencies (Whang et al., 2003; Chak and Leung, 2004; Caplan, 2007; Ebeling-Witte et al., 2007; Hardie and Tee, 2007; Thatcher et al., 2008; Kim and Davis, 2009; Pontes et al., 2014), and social isolation/lack of social support (Morahan-Martin and Schumacher, 2003; Caplan, 2005) in the development of GIA. However, we suggested that the influence of those person’s primary characteristics and cognitions on the development of an addictive use of the Internet should be mediated by certain Internet-related cognitions, in particular Internet use expectancies (Turel et al., 2011; Xu et al., 2012; Lee et al., 2014), and certain strategies to cope with everyday life requirements or daily hassles (Tang et al., 2014; Tonioni et al., 2014). In the third section of the model, as a consequent behavior, if the user goes online and receives reinforcement in terms of dysfunctional coping with problems or negative mood and the person expects that Internet use will distract them from problems or negative feelings, then the more likely they will turn to the Internet to escape those feelings evidenced by a loss of control, poor time management, cravings, and increased social problems. The role of reinforcement and conditioning processes has been described well in the literature on the development and maintenance of substance related disorders (e.g., Robinson and Berridge, 2001, 2008; Kalivas and Volkow, 2005; Everitt and Robbins, 2006). We have also argued that the positive and negative reinforcement of coping style and Internet use expectancies successively result in a loss of cognitive control over the Internet use, which is mediated by prefrontal (executive) functioning (Brand et al., 2014).

Although this model fits well with previous literature on key findings with regards to psychological mechanisms behind IA (see overviews by Kuss and Griffiths, 2011a,b; Griffiths, 2012) and also with very recent neuropsychological and neuroimaging correlates of GIA and distinct types of SIA (Kuss and Griffiths, 2012; Brand et al., 2014), this model still needs empirical evidence in terms of incremental validity. In this study, we aimed at translating the hypotheses summarized in the theoretical model on GIA outlined above into a statistical model on latent variables level and tested the predictor and mediator effects on the severity of GIA symptoms using a large scale Internet population. Using validated psychological and personality measures, we first assessed a persons’ core characteristics in predicting an excessive and addictive use of the Internet in a generalized way. Using a validated measure of coping and a newly developed measure of Internet use expectancies, we tested if poor coping skills and Internet use expectancies (such as using the Internet to escape negative feelings or unpleasant situations) mediate the link between person’s core characteristics and symptoms of GIA.

## MATERIALS AND METHODS

### THE OPERATIONALIZED MODEL

**FIGURE 1:**
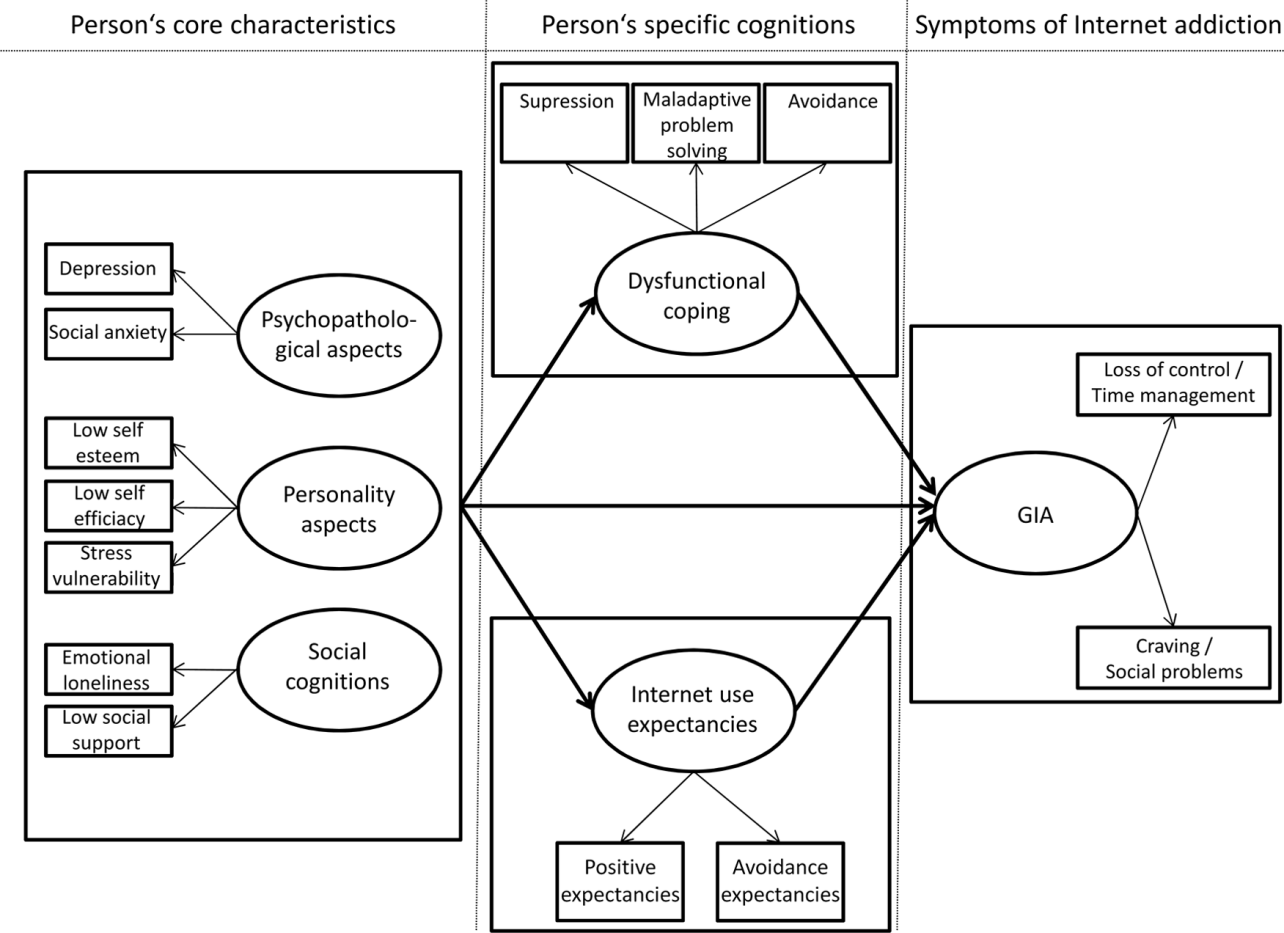
**The operationalized model, including main assumptions of the theoretical model on GIA, on latent dimension**.

We first translated the theoretical model described in the introduction and illustrated in the article by Brand et al. (2014) into a testable and operationalized statistical model. For each of the dimensions mentioned in the theoretical model, we chose at least two manifest variables to build a structural equation model (SEM) on latent level. For each variable, we then used a specific scale (each consisting of several items, see description of the instruments below) to operationalize the manifest variables. This operationalized model as SEM on latent level is shown in **Figure 1**.

### SUBJECTS

Using a comprehensive online survey, we had 1148 respondents. After exclusion of 129 participants due to incomplete data in the psychometric scales, the final sample consisted of *N* = 1019. The participants were recruited by advertisements, Internet platforms (Facebook account of the team General Psychology: Cognition), e-mail lists to students of the University of Duisburg-Essen, and via flyers in local pubs and bars as well as word-of-mouth recommendations. The advertisements, e-mails, and flyers included a statement that participants can take part in a raﬄe having the chance to win one of the following items: (1) iPad, (2) iPad mini, (3) iPod nano, (4) iPod shuﬄe, 20 Amazon gift cards (50 Euros each). The study was approved by the local ethics committee.

Mean age of the final sample was 25.61 years (SD = 7.37). The sample included 625 (61.33%) females and 385 (37.78%) males (nine volunteers did not answer this question). With respect to private life situation, 577 participants (56.62%) lived in a relationship or were married and 410 (40.24%) indicated to not have a current relationship (32 participants did not respond to this question). At time of assessment, 687 participants (67.42%) were students, 332 participants (32.58%) had a regular job (with our without academic background). Of the whole sample, 116 participants (11.4%) fulfilled criteria for problematic Internet use [cut-off >30 in the short Internet Addiction Test (s-IAT), see description of the instrument below] and 38 participants (3.7%) for a pathological use of the Internet (>37 in the s-IAT). Mean time spent on the Internet was 972.36 min/week (SD = 920.37). Of the whole sample, 975 individuals used social networking/communication sites (*M*_min/week_ = 444.47, SD = 659.05), 998 individuals (97.94%) searched information on the Internet (*M*_min/week_ = 410.03, SD = 626.26), 988 individuals (96.96%) used shopping sites (*M*_min/week_ = 67.77, SD = 194.29), online games were used by 557 participants (54.66%, *M*_min/week_ = 159.61, SD = 373.65), online gambling was done by 161 participants (15.80%, *M*_min/week_ = 37.09, SD = 141.70), and cybersex was used by 485 individuals (47.60%, *M*_min/week_ = 66.46, SD = 108.28). Regarding the use of multiple Internet applications, 995 participants (97.64%) reported to use three or more of the Internet applications mentioned above on a regular basis.

### INSTRUMENTS

#### Short Internet Addiction Test (s-IAT)

Symptoms of IA were assessed with the German short version of the Internet Addiction Test (Pawlikowski et al., 2013), which is based upon the original version developed by Young (1998). In the short-version (s-IAT), 12 items have to be answered on a five-point scale ranging from 1 (= never) to 5 (= very often) resulting in sum scores ranging from 12 to 60, whereas scores >30 indicates a problematic Internet use and score >37 indicates pathological Internet use (Pawlikowski et al., 2013). The s-IAT consists of two factors: loss of control/time management and craving/social problems (each having six items). Although the 12 items load on two factors in both exploratory and confirmatory factor analysis (CFA; Pawlikowski et al., 2013), they capture the key symptoms of IA, as for example described in the components model by (Griffiths, 2005). The first subscale “loss of control/time management” assesses how strong a person suffers from time management problems in everyday life due to his/her Internet use (e.g., “How often do you neglect household chores to spend more time online?” and “How often do you lose sleep due to being online late at night?”). Items of this subscale also assess negative consequences caused by the excessiveness of the Internet use (e.g., “How often do your grades or school work suffer because of the amount of time you spend online?”). It is also measured if the subjects experience loss of control over their Internet use and if they had tried to reduce their Internet use and failed (e.g., “How often do you find that you stay online longer than you intended?” and “How often do you try to cut down the amount of time you spend online and fail?”). All items do not measure the time spent online, but whether or not individuals experience a loss of control regarding their Internet use and problems in everyday life as a result of their Internet use. The second subscale “craving/social problems” measures effects of excessive Internet use on social interactions and preoccupation with the medium (e.g., “How often do you feel preoccupied with the Internet when oﬄine, or fantasize about being online?”). Items of this subscale also assess inter-personal problems (e.g., How often do you snap, yell, or act annoyed if someone bothers you while you are online?”) and mood regulation (e.g., “How often do you feel depressed, moody, or nervous when you are oﬄine, which goes away once you are back online?). All items include the terms “Internet” or “online” in general without focusing on a certain application. In the instruction, the participants were informed that all questions relate to their general use of the Internet including all applications used.

The s-IAT has good psychometric properties and validity (Pawlikowski et al., 2013). In our sample, internal consistency (Cronbach’s α) was 0.856 for the whole scale, 0.819 for the factor loss of control/time management, and 0.751 for the factor craving/social problems.

#### Brief Symptom Inventory – subscale depression

Symptoms of depression were assessed with the German version (Franke, 2000) of the subscale depression of the Brief Symptom Inventory (Boulet and Boss, 1991; Derogatis, 1993). The scale consists of six items assessing depressive symptoms for the last 7 days. Answers have to be given on a five-point scale ranging from 0 (= not at all) to 4 (= extremely). Internal consistency (Cronbach’s α) in our sample was 0.858.

#### Brief Symptom Inventory – subscale interpersonal sensitivity

Symptoms of social anxiety and interpersonal sensitivity were assessed with the German version (Franke, 2000) of the subscale interpersonal sensitivity of the Brief Symptom Inventory (Boulet and Boss, 1991; Derogatis, 1993). The scale consists of four items and answers have to be given on a five-point scale ranging from 0 (= not at all) to 4 (= extremely). Internal consistency (Cronbach’s α) in our sample was 0.797.

#### Self-Esteem Scale

Self-esteem was assessed by the Self-Esteem Scale (Rosenberg, 1965). We here used the modified German version (Collani and Herzberg, 2003), which consists of ten items. The answers have to be given on a four-point scale ranging from 0 (= strongly disagree) to 3 (= strongly agree). Internal consistency (Cronbach’s α) in our sample was 0.896.

#### Self-Efficacy Scale

Self-efficacy was assessed by the Self-Efficacy Scale (Schwarzer and Jerusalem, 1995), which consists of 10 items. Answers have to be given on a four-point scale ranging from 1 (= not true) to 4 (= exactly true). Internal consistency (Cronbach’s α) in our sample was 0.863.

#### Trier Inventory for Chronic Stress

Stress vulnerability was measured by the screening version of the Trier Inventory for Chronic Stress (TICS; Schulz et al., 2004). The screening contains 12 items about stress exposure in the last 3 months. Each statement has to be answered on a five-point scale ranging from 0 (= never) to 4 (= very often). Internal consistency (Cronbach’s α) in our sample was 0.908.

#### Loneliness scale

The short version of the Loneliness Scale (De Jong Gierveld and Van Tilburg, 2006) was used to measure feelings of loneliness (subscale emotional loneliness, three items) and perceived social support (social support subscale, three items). All statements have to be answered on a five-point scale from 1 (= no!) to 5 (= yes!). Internal consistency (Cronbach’s α) in our sample was 0.765 for the subscale emotional loneliness and 0.867 for the subscale social support.

#### Brief COPE

The Brief COPE (Carver, 1997) measures coping style in several different subdomains. We here used three subscales of the German version (Knoll et al., 2005): denial, substance use, and behavioral disengagement. Each subscale was represented by two items, which had to be answered on a four-point scale ranging from 1 (= I haven’t been doing this at all) to 4 (= I’ve been doing this a lot). Internal consistency (Cronbach’s α) in our sample was 0.561 for the subscale denial, 0.901 for the subscale substance use, and 0.517 for the subscale behavioral disengagement. Given that the scales consist of only two items and given that the instrument has been used in several validation studies including reports on re-test reliability, we consider the reliability as acceptable.

#### Internet Use Expectancies Scale

To assess Internet use expectancies, we developed a new scale consisting – in the first version – of 16 items. The items reflect some core motivating factors as, for example, reported by Xu et al. (2012) and also by Yee (2006). The items were assigned *a priori* to two scales (each having eight items): Internet use expectancies reflecting positive reinforcement (e.g., “I use the Internet to experience pleasure”) and those reflecting negative reinforcement (e.g., “I use the Internet to distract from problems”). All answers were given on a six-point scale ranging from 1 (= completely disagree) to 6 (= completely agree). On the basis of the data we have collected in this study (*N* = 1019), we conducted an exploratory factor analysis (EFA). Horn’s (1965) parallel analysis and the minimum average partial (MAP) test (Velicer, 1976) were used to determine the appropriate number of factors. This procedure resulted in a stable two-factor solution. An EFA with principal component analysis and varimax rotation was then conducted to assess the structure of the Internet Use Expectancies Scale (IUES). Results of the EFA concluded with a final 8-item version of the IUES with the two-factor structure remains (**Table 1**). With these two factors, we observed a variance explanation of 63.41%. The first factor contains four items with high loadings on the main factor (>0.50) and low loadings on the other factor (<0.20) and relates to positive expectancies, so we named this factor “positive expectancies.” The second factor consists of four items with high loadings on the main factor (>0.50) and low loadings on the other factor (<0.20), and all items related to Internet use to avoid or reduce negative feelings or thoughts, so we named this factor “avoidance expectancies.” Both factors have good reliability (“positive expectancies”: Cronbach’s α = 0.832 and “avoidance expectancies” Cronbach’s α = 0.756). The two factors were correlated significantly (*r* = 0.496, *p* < 0.001) with a moderate effect (Cohen, 1988).

**Table 1:** Factor loadings and reliabilities of the two factors of the IUES, means of the rated items and the item numbers.

Item number*	Item	Factor		*M*	(*SD*)
		1	2			
**(1) Factor: positive expectancies**
Q1	To experience pleasure	**0.774**	-0.089	3.85	(1.22)
Q3	To have fun	**0.731**	-0.122	4.40	(1.11)
Q7	To gain positive emotions	**0.728**	0.158	3.27	(1.30)
Q5	To feel good	**0.673**	0.191	3.08	(1.29)
**(2) Factor: avoidance expectancies**
Q2	To distract from problems	-0.121	**0.870**	2.45	(1.35)
Q6	To escape from reality	-0.035	**0.765**	2.07	(1.25)
Q4	To avoid loneliness	0.107	**0.531**	2.40	(1.39)
Q8	To avoid annoying duties	0.065	**0.501**	3.11	(1.45)
	**Reliability (Cronbach’s** α**)**	**0.832**	**0.756**		

All items started with “I use the Internet …”

*Item number now adapted for the 8-item version. Numbers also represent their relative position in the 16-item version.

To ensure the factorial structure of the instrument, we assessed an additional sample of 169 subjects (mean age = 21.66, SD = 2.69; 106 females) for applying a CFA. The CFA was done with MPlus (Muthén and Muthén, 2011). For the evaluation of model fits, we applied standard criteria (Hu and Bentler, 1995, 1999): The standardized root mean square residual (SRMR; values below 0.08 indicate good fit with the data), comparative fit indices (CFI/TLI; values above 0.90 indicate a good fit, values above 0.95 an excellent fit), and root mean square error of approximation (RMSEA; “test of close fit”; a value below 0.08 with a significance value below 0.05 indicates acceptable fit). The CFA confirmed the two-factor solution for the IUES with good to excellent fit parameters: The RMSEA was 0.047, the CFI was 0.984, the TLI was 0.975, and the SRMR was 0.031. The χ^2^ test was not significant, χ^2^ = 24.58, *p* = 0.137 indicating that the data did not deviate significantly from the theoretical model (two factors solution, as shown in **Table 1**).This sample was collected for the CFA, only. The data were not included in the further analyses.

### STATISTICAL ANALYSES

Statistical standard procedures were carried out with SPSS 21.0 for Windows (IBM SPSS Statistics, released 2012). Pearson correlations were calculated to test for zero-order relationships between two variables. To control the data for outliers, we created a normally distributed random variable with the same mean standard deviation as we found in the s-IAT (overall score). This random variable should theoretically be unrelated to all variables of interest, if the correlations were not influenced by outliers in the data. All correlations with the random variable were very low, *r*s < 0.049, indicating that there were no substantially influential outliers in any of the scales in the final sample (*N* = 1019). Additionally scatterplots between the variables were controlled visually. Again, no extreme outliers were found. Therefore, the analyses were performed with all subjects.

The SEM analysis was computed with MPlus 6 (Muthén and Muthén, 2011). There were no missing data. Before testing the full model, the fits of the latent dimensions were also tested using CFA in MPlus. For both, SEM and CFA, maximum likelihood parameter estimation was applied. For the evaluation of model fits, we applied the standard criteria (Hu and Bentler, 1995, 1999) as already described in the section before. For applying mediator analysis it was required, according to Baron and Kenny (1986), that all variables included in the mediation should correlate with each other. We also used moderated regressions for analyzing potential moderator effects as additional analyses for an alternative conceptualization of the coping concept.

## RESULTS

### DESCRIPTIVE VALUES AND CORRELATIONS

The samples’ mean scores in the s-IAT and all other scales applied can be found in **Table 2**. The mean s-IAT score of *M* = 23.79 (SD = 6.69) is quite comparable with the score reported by Pawlikowski et al. (2013) for a sample of 1820 subjects of the general population (the mean s-IAT score was *M* = 23.30, SD = 7.25). The bivariate correlations between the s-IAT (sum score) and the scores in the questionnaires and scales administered are shown in **Table 3**.

**Table 2:** Mean scores of the scales applied.

*N* = 1019	*M*	*(SD)*
s-IAT (sum score)	23.79	(6.69)
BSI depression	0.65	(0.71)
BSI interpersonal sensitivity	0.82	(0.79)
Self-esteem scale	2.22	(0.52)
Self-efficacy scale	2.97	(0.42)
TICS stress vulnerability	1.64	(0.75)
Emotional loneliness	2.27	(0.86)
Social support	4.07	0.88)
COPE denial	1.49	(0.61)
COPE substance use	1.36	(0.65)
COPE behavioral disengagement	1.40	(0.50)
IUES positive expectancies	3.65	(1.01)
IUES avoidance expectancies	2.51	(1.04)

**Table 3:** Bivariate correlations between the s-IAT (sum score) and the scores in the questionnaires administered.

		1 s-IAT	2	3	4	5	6	7	8	9	10	11	12	13
												
**2**	BSI depression	0.33***											
**3**	BSI interpersonal sensitivity	0.32***	0.73***										
**5**	Self-esteem scale	-0.30***	-0.64***	-0.58***									
**6**	Self-efficacy scale	-0.25***	-0.45***	-0.44***	0.65***								
**7**	TICS stress vulnerability	0.41***	0.57***	0.58***	-0.56***	-0.46***							
**8**	Emotional loneliness	0.32***	0.63***	0.59***	-0.58***	-0.41***	0.45***						
**9**	Social support	-0.20***	-0.45***	-0.43***	0.41***	0.27***	-0.26***	-0.58***					
**10**	COPE denial	0.23***	0.30***	0.28***	-0.25***	-0.21***	0.35***	0.22***	-0.12***				
**11**	COPE substance use	0.25***	0.26***	0.22***	-0.15***	-0.07*	0.23***	0.14***	-0.12***	0.25***			
**12**	COPE behavioral disengagement	0.27***	0.32***	0.25***	-0.29***	-0.24***	0.29***	0.29***	-0.21***	0.33***	0.21***		
**13**	IUES positive expectancies	0.43***	0.18***	0.17***	-0.15***	-0.13***	0.18***	0.16***	-0.08**	0.10***	0.09***	0.12***	
**14**	IUES avoidance expectancies	0.55***	0.41***	0.39***	-0.39***	-0.29***	0.40***	0.40***	-0.22***	0.17***	0.21***	0.20***	0.50***

*p ≤ 0.05; **p ≤ 0.01; ***p ≤ 0.001.

### LATENT DIMENSIONS OF THE PROPOSED MODEL IN CONFIRMATORY FACTOR ANALYSIS

In order to systematically test the proposed theoretical model, we first analyzed the factor model, which means that it was tested whether the latent dimensions are acceptably represented by the manifest variables. Therefore, CFA was performed with the six latent dimensions (one dependent dimension, three predictor dimensions, two mediator dimensions). The RMSEA was 0.066 with *p*< 0.001, the CFI was 0.951, the TLI was 0.928 and the SRMR was 0.041, indicating a good model fit.

The first latent dimension “symptoms of GIA” was represented well by the scores in the two factors of the s-IAT (loss of control/time management and craving/social problems) as intended. The first predictor variable “psychopathological symptoms” was significantly represented by the two subscales of the BSI (depression and interpersonal sensitivity). The dimension “personality aspects” was well represented by the three hypothesized manifest variables (self-efficacy, self-esteem, and stress vulnerability) and the last predictor dimension “social cognitions” was represented well by the two subscales of the loneliness scale (emotional loneliness and social support). Results showed that the first hypothesized mediator dimension “coping” was well represented by the three subscales of the COPE (denial, substance abuse, and behavioral disengagement) and the second mediator dimension “Internet use expectancies” was well represented by the two IUES factors (positive expectancies and avoidance expectancies).

Overall, the CFA indicated that the latent dimensions are represented acceptably by the manifest variables. Only in the dimension coping the scale substance abuse has a weaker factor loading (β = 0.424) but still significant (*p* < 0.001) and therefore sufficient, given that the overall model fitted well with the data. All factor loadings and standard errors are shown in **Table 4**.

**Table 4:** Coefficients of the manifest variables’ loadings on the latent dimensions, tested with CFA in MPlus.

Latent dimension	Manifest variables	β	*SE*
GIA	s-IAT factor loss of control/time management	0.804***	0.020
	s-IAT factor craving/social problems	0.746***	0.021
Psychopathology	BSI depression	0.882***	0.011
	BSI interpersonal sensitivity	0.826***	0.013
Personality	Self-esteem	0.862***	0.015
	Self-efficacy	0.694***	0.020
	TICS stress vulnerability	-0.699***	0.020
Social aspects	Emotional loneliness	-0.917***	0.019
	Social support	0.635***	0.023
Coping	COPE denial	0.563***	0.033
	COPE substance abuse	0.424***	0.035
	COPE behavioral disengagement	0.569***	0.033
Internet use expectancies	Positive expectancies	0.568***	0.027
	Avoidance expectancies	0.873***	0.026

***p ≤ 0.001.

### THE FULL STRUCTURAL EQUATION MODEL

**FIGURE 2:**
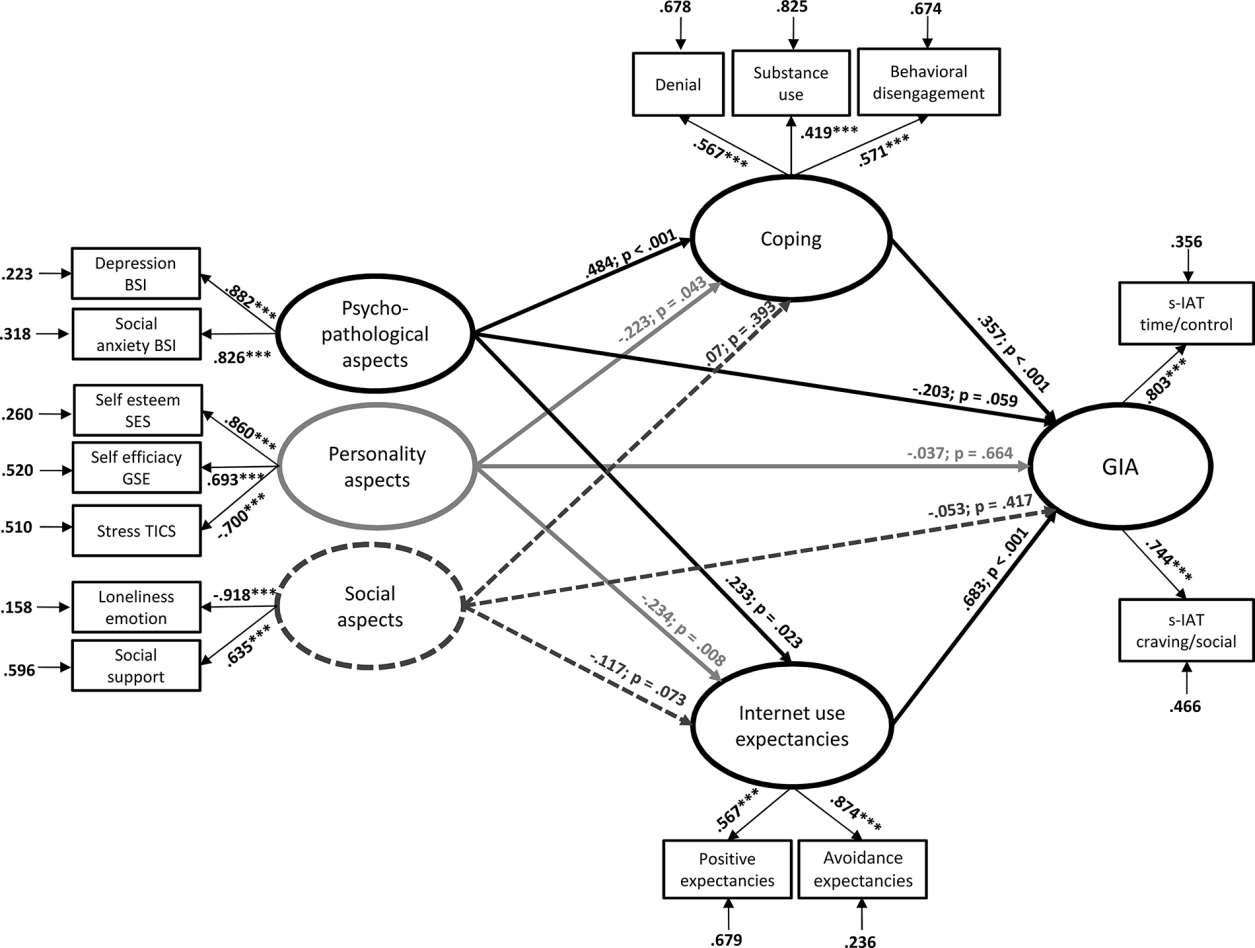
**Results of the structural equation model including factor loadings of the latent dimensions, β-weights, *p*-values, and residuals.** ***p < 0.001.

The proposed theoretical model on latent dimension with GIA as dependent variable (modeled by the two s-IAT factors) yielded a good fit with the data. The RMSEA was 0.066 with *p*< 0.001, the CFI was 0.95, the TLI was 0.93, and the SRMR was 0.041. The χ^2^ test was significant, χ^2^ = 343.89, *p*< 0.001, which is normal given the large sample size. However, the χ^2^ test for the baseline model was also significant with an extensively higher χ^2^ value, χ^2^ = 5745.35, *p*< 0.001. In summary, the data fitted well with the proposed theoretical model. Overall, the large proportion of 63.5% of the variance in GIA was significantly explained by the full SEM (*R*^2^ = 0.635, *p*< 0.001). The model and all direct and indirect effects are shown in **Figure 2**.

All three direct effects of the predictors on GIA were not significant (**Figure 2**). But note that the direct effect of the latent variable psychopathological aspects slightly failed to reach significance with *p* = 0.059. Here, it has to be considered that the β-weight was negative, indicating that – in case one would interpret the marginally significant direct effect – higher depression and social anxiety go hand in hand with lower symptoms of GIA if the indirect effect from psychopathological aspects over the two mediator variables (coping and Internet use expectancies) are partialized. The direct effects from the two latent predictor variables psychopathological aspects and personality on both latent mediator variables coping and Internet use expectancies were significant. By contrast, the direct effects from the latent variable social cognitions on both coping and Internet use expectancies were not significant, which means that these effects were not significant when controlled for the effects of the other two latent dimensions. However, the effects from social cognitions to Internet use expectancies slightly failed to reach significance with *p* = 0.073. The direct effects from coping to GIA (*p* < 0.001) and from Internet use expectancies (*p* < 0.001) were significant with strong effect sizes.

The indirect effect from psychopathological aspects over coping to GIA was significant (β = 0.173, SE = 0.059, *p* = 0.003). Also the indirect effect from psychopathological aspects over Internet use expectancies to GIA was significant (β = 0.159, SE = 0.072, *p* = 0.027). The indirect effect from personality aspects over coping to GIA was also significant (β = –0.08, SE = 0.041, *p* = 0.05), but the effect size was very small. The indirect effect from personality aspects over Internet use expectancies to GIA was significant (β = –0.160, SE = 0.061, *p* = 0.009). Both indirect effects from social cognitions over coping (β = 0.025, SE = 0.030, *p* = 0.403) and social cognition over Internet use expectancies (β = –0.08, SE = 0.045, *p* = 0.075) to GIA were not significant. The model with all factor loadings and *β*-weights is shown in **Figure 2**. The latent dimension psychopathological aspects was significantly correlated with the latent dimension personality aspects (*r* = -0.844, *p* < 0.001) and with the latent dimension social cognitions (*r* = –0.783, *p* < 0.001). Also, the two latent dimensions personality aspects and social cognitions were correlated (*r* = 0.707, *p* < 0.001).

### ADDITIONAL ANALYSES

The model described was the theoretically argued one and consequently that which we tested first. However, we afterward tested some additional models or parts of the model separately in order to better understand the underlying mechanisms of GIA in more detail. The first issue we addressed was the effect of psychopathology on GIA, because we found it interesting that the direct effect, albeit not significant, was negative in the SEM (see **Figure 2**), although on the bivariate level, the correlations were positive. The simple model with psychopathological aspects (represented by BIS depression and BSI social anxiety) as predictor and GIA (represented by the two s-IAT factors) as dependent variable had a good model fit (all fit indices are better than acceptable) and the effect was positive (β = 0.451, *p* < 0.001). We also calculated the model without the two mediators, meaning that psychopathological aspects, personality aspects, and social aspects served as direct predictors and GIA was the dependent variable (all variables on latent level with the same variables used in the whole SEM, see **Figure 2**). The model without mediators had also good fit indices (with one exception: the RMSEA was with 0.089 a little bit high) and the direct effects on GIA (the two s-IAT factors) were: effect of psychopathological aspects on GIA β = 0.167, *p* = 0.122; effect of personality aspects on GIA β = –0.223, *p* = 0.017; and effect of social aspects on GIA β = –0.124, *p* = 0.081. Note that the effect of psychopathological aspects on GIA is still positive in this model (but not significant) when the effect is controlled for the effects of personality and social aspects. Taken together, the results of the overall SEM speaks for a full mediation of the effect of psychopathological aspects on GIA by the two mediators (coping and expectancies), which is further emphasized by the two additional analyses showing that the positive effect on a bivariate level and in the simple model is reduced by the inclusion of further variables as predictors.

We have theoretically conceptualized coping as a mediator (Brand et al., 2014). However, one may also argue that coping does not mediate the effect of psychopathological aspects, but act as a moderator. To ensure that the conceptualization of coping as a mediator instead of a moderator is appropriate, we additionally calculated some moderator analyses using moderated regression analyses. When, for example, using psychopathological aspects as predictor, coping as moderator, and s-IAT (sum score) as dependent variable, both psychopathological aspects (β = 0.267) and coping (β = 0.262) explain the variance in the s-IAT significantly (both *p* < 0.001), but their interaction does not significantly add variance explanation (changes in *R*^2^ = 0.003, *p* = 0.067, β = -0.059) and the increment of the moderator effect is almost zero (0.3%).

We also considered age and gender as potential variables which may have an effect on the structure of the model. To test this, we first calculated the bivariate correlations between age and all other variables resulting in very low correlations. There was only one correlation with *r* = 0.21 (age and avoidance expectancies), which is still a low effect (Cohen, 1988), and all other correlations had effects between *r* = 0.016 and *r* = 0.18 with most being *r* < 0.15 and *r* < 0.10. The correlation between age and the s-IAT was also very low with *r* = –0.14 (although significant at *p*< 0.01, which is clear in such a large sample). In summary, the requirements for including age into the mediation model were not fulfilled (Baron and Kenny, 1986) and we decided to not include age into an additional model. With respect to gender, we compared groups’ mean scores of all scales used and found only one meaningful group difference (BSI social anxiety, females had higher scores with a low effect of *d* = 0.28, all other effects were lower than 0.28, the effect for the s-IAT score was *d* = 0.19). We nevertheless tested whether the model structure is different for women and men using mean structure analysis in the SEM analysis. This means that we tested if the SEM (see **Figure 2**) is equal for male and female participants. The H0 of this test is: theoretical model = model for the group “men” = model for the group “women.” The fit indices were all acceptable indicating that the structure of the relationships was not significantly different for men and women. The RMSEA was 0.074 with *p*< 0.001, the CFI was 0.93, the TLI was 0.91, and the SRMR was 0.054. The χ^2^ test was significant, χ^2^ = 534.43, *p*< 0.001, which is normal given the large sample size. However, the χ^2^ test for the baseline model was also significant with an extensively higher χ^2^ value, χ^2^ = 5833.68, *p*< 0.001. The contribution to the χ^2^ of the tested model by men and women were comparable (χ^2^ contributions by women = 279.88, χ^2^ contributions by men = 254.55). Although the overall structure of the model is not significantly different for men and women, we inspected the simple path and found three differences. The path from personality aspects to coping was significant in men (β = –0.437, *p* = 0.002), but not in women (β = –0.254, *p* = 0.161) and the effect from personality aspects on expectancies was significant in men (β = -0.401, *p* = 0.001), but not in women (β = –0.185, *p* = 0.181). In addition, the effect from psychopathological aspects on expectancies was significant in women (β = 0.281, *p* = 0.05), but not in men (β = 0.082, *p* = 0.599). All other effects and the representation of the latent dimensions were not different between men and women and also not different from the overall model illustrated in **Figure 2**. In summary, the whole model tested is valid for men and women, although the negative effect of personality aspects on coping and expectancies is more present in men compared to women and the effect from psychopathological aspects on expectancies is present in women, but not in men.

## DISCUSSION

We have introduced a new theoretical model on the development and maintenance of an addictive use of the Internet (Brand et al., 2014), which is based on the main arguments by Davis (2001) who first suggested a differentiation between a generalized overuse of the Internet (GIA) and a specific addiction to certain Internet applications (SIA). In the current study, we translated the theoretical model on GIA into an operationalized model on latent level and statistically tested the SEM using an online survey on an Internet population of 1019 respondents. We found an overall good model fit with the data and the hypothesized SEM, which represents the main facets of the theoretical model and explained 63.5% of the variance of GIA symptoms as measured by the s-IAT (Pawlikowski et al., 2013).

The model is the first to tie together elements associated with IA such as depression, social anxiety, low self-esteem, low self-efficacy, and higher stress vulnerability. Based on the emphasis of cognitions related to developing IA and to addictive behavior in general (Lewis and O’Neill, 2000; Dunne et al., 2013; Newton et al., 2014), the model investigates if two mediator variables (coping styles and Internet use expectancies) impact the direct effects of the predictor variables (psychopathology, personality, and social cognitions) on the development of GIA. Results show that both coping styles and Internet use expectancies play a significant role.

All variables (predictors and mediators) included in the model were significantly correlated with the s-IAT score on a bivariate level. This is basically consistent with previous research on bivariate relationships between symptoms of IA and personality aspects, psychopathological symptoms and other person variables, as mentioned in the Introduction. However, in the SEM analysis, all direct effects of the three main predictors (on latent dimension) were no longer significant when including the hypothesized mediators into the model. This means that psychopathological aspects (depression, social anxiety), personality aspects (self-esteem, self-efficacy, and stress vulnerability) as well as social cognitions (emotional loneliness, perceived social support) do not impact symptoms of GIA directly, but that their influence is mediated by either a dysfunctional coping style, or Internet use expectancies, or both. However, psychopathological aspects and personality aspects significantly predict both dysfunctional coping style and Internet use expectancies. Social cognitions, however, are not significantly related to coping and expectancies, when their relative impact is controlled for the effects of psychopathological and personality aspects (but note that the three predictor latent dimensions were correlated significantly and that the effect from social cognitions to Internet use expectancies slightly failed to reach significance). The direct effects of both coping style and expectancies on symptoms of GIA were significant. In summary, the current study, although with a non-clinical population, not only confirms the previous findings on the relevance of coping style and dealing with stressful life events (Kardefelt-Winther, 2014; Tang et al., 2014; Tonioni et al., 2014) as well as Internet use expectancies (Turel and Serenko, 2012; Xu et al., 2012; Lee et al., 2014) for developing or maintaining symptoms of GIA, but explicitly highlights the role of coping and expectancies as mediators in the process underlying GIA.

The model was tested with a large online population. Model must be tested with clearly defined clinical samples, such as treatment-seeking individuals. The meaning of the model would be more robust with a clinical population to draw more accurate clinical implications. Although 11.3% of the sample reported a problematic Internet use and 3.7% described themselves as having an addictive Internet use, this study is considered only an initial look to see if the model works and draws statistical inferences that could potentially have clinical relevance. However, as a new model with statistical significance using a variety of psychological and personality tests on online users, a few clinical implications, which may inspire future research, can be made with caution.

First, individuals with dysfunctional coping to deal with problems in their life and who have expectancies that the Internet can be used to increase positive or reduce negative mood may be more likely to develop GIA. Moreover, the effects of psychopathological aspects on both dysfunctional coping and Internet use expectancies were positive indicating that higher symptoms of depression and social anxiety can increase the risk for dysfunctional coping strategies and also for the expectancies that the Internet provides help for dealing with stress or negative mood. Only when these processes act in concert, meaning the combination of psychopathological symptoms and coping/expectancies, the probability of using the Internet addictively seems to increase.

Secondly, although the number of studies addressing treatment of GIA is limited, the meta-analysis published by Winkler et al. (2013) argues that cognitive-behavioral therapy is the method of choice. This is particularly based upon the analysis of treatment effects on time spent online, depression, and anxiety symptoms. In fact, cognitive-behavioral therapy for IA (CBT-IA; Young, 2011a) has been identified as the most prevalent form of treating IA (Cash et al., 2012). Within cognitive-behavioral treatment of GIA proposed by Young (2011a), individual characteristics as well as coping and Internet use expectations have already been hypothesized to be relevant within the treatment of GIA, but the empirical evidence was very sparse (e.g., Young, 2013).

The findings presented in this study provide one further source of evidence to show that cognitive-behavioral therapy and CBT-IA can work to treat IA. The person’s specific cognitions (coping style and Internet use expectancies) mediate the impact of psychopathological symptoms (depression, social anxiety), personality traits, and social cognition (loneliness, social support) on GIA symptoms. Using cognitive therapy, an emphasis in assessment should include identifying dysfunctional cognitions to be addressed. That is, upon examination, clinicians should examine Internet use expectancies to understand the needs of the client and what ways the client believes the Internet may help to satisfy.

Alternatively, findings also suggest that therapy should address maladaptive cognitions associated with dysfunctional use of the Internet. These findings confirm earlier studies that showed maladaptive cognitions such as overgeneralization, avoidance, suppression, magnification, maladaptive problem solving, or negative self-concepts are associated with addictive Internet use (Young, 2007). A clinical implication of these findings is that therapy should apply cognitive restructuring and reframing to combat thoughts that lead to addictive use of the Internet. For instance, a patient suffering from GIA may have signs of social anxiety and shyness and therefore a few friends and also trouble with others at school. She may then think that communicating with other people via social networking sites gratifies her social need without having the scary situational aspects of a “real” social interaction. In addition, she may have the expectancy that also playing an online game may distract her from the problems at school and that buying online or searching information on the Internet may reduce the feelings of loneliness. Therapy would focus her on seeing alternative places at school or in private life where she can build up esteem and gratify social needs. If she stops justifying that the social networking sites, games and shopping sites are the only places she feels good about her life and she finds other healthier outlets, the less reliant she will be on the different Internet applications. Knowing the role that cognitions play in the development of GIA, cognitive therapy can help clients restructure the assumptions and interpretations that keep them online. Again, these potential clinical implications of the study’s results must be treated with caution, since they must be replicated in a treatment-seeking, clinical sample.

From a broader perspective, however, these findings gain insights into how therapists can specifically apply CBT-IA to Internet-addicted patients. Behavior modification can help clients develop and adapt new and more functional coping strategies in order to deal with daily hassle. Therapy needs to focus on helping clients find healthier ways of coping than turning to the Internet. A major component of CBT-IA is behavior therapy to help clients cope with underlying issues contributing to IA, specific or generalized (Young, 2011a, 2013). The findings suggest that improving coping skills would reduce the need to go online for clients. Although studied in a sample of the general population, we believe that the finding that coping and expectancies are mediators in the development and maintenance of GIA contribute to a better understanding of the mechanisms of GIA and that they likely have some treatment implications, as mentioned above. Another aspect that was not focused in the current study is the role of prefrontal cortex integrity. Efficacy of CBT-IA may also depend upon the patient’s prefrontal functioning, because strengthening cognitive control of the Internet use in the course of the therapy is most likely related to executive functions and other higher-order cognitive processes. This is important to address in future studies, because most recently there have been a couple of articles published showing that prefrontal cortex functions are likely reduced in patients with IA (see overview in Brand et al., 2014).

In our sample, age was inversely correlated with symptoms of GIA, but with a very low effect size (explaining 1.96% of the variance, only). Considering recent articles on Internet use in older individuals (e.g., Eastman and Iyer, 2004; Vuori and Holmlund-Rytkönen, 2005; Campbell, 2008; Nimrod, 2011), one may certainly except age effects on several aspects of using the Internet, such as using motives and the way elderly experience fun and satisfaction on the Internet. Given that elderly people also have a higher chance to develop executive dysfunctions due to prefrontal cortex changes with increasing age (Alvarez and Emory, 2006), which are also linked to decision-making reductions (Brand and Markowitsch, 2010), one may speculate that those older individuals with executive reductions, who experience a large amount of pleasure on the Internet may develop GIA. However, this is not represented by our data, since our sample did not include older subjects. Future studies may investigate the specific vulnerability factors linked to the risk of GIA in older adults.

Gender did not affect the overall structure of the model. In previous articles, gender effects have been found for specific types of IA, such as online gaming (e.g., Ko et al., 2005) and particularly cybersex (Meerkerk et al., 2006; Griffiths, 2012; Laier et al., 2013, 2014), but it has also been argued that both genders are generally at risk for developing an addictive use of the Internet (Young et al., 1999, 2011). In our study, the effects of gender on GIA, as measured by the s-IAT, was very low (*d* = 0.19, see results), indicating that at least in a general population both gender are equally at risk for developing GIA. Although gender did not affect the general data structure in the SEM, there were some differences between men and women with respect to three direct effects from predictor variables to the mediators. As summarized in the results section, psychopathological aspects had an effect on expectancies in women, not in men, in the negative effect of personality aspects on coping and expectancies is more present in men than in women. These effects fit with the literature on gender differences with respect to depression and social anxiety (Sprock and Yoder, 1997; Moscovitch et al., 2005), as well as self-esteem and self-efficacy (Huang, 2012). However, the facets which are the focus of the study, namely the mediation effects of coping and expectancies and their importance for GIA were not affected by gender (see results of the mean structure analysis). So independently of how gender may influence social anxiety, depression or some personality aspects, coping and expectancies should be considered in CBT-IA in both genders.

Finally, there are several limitations of this study. It is a newly developed model that needs further testing on a clinical population to fully see its clinical efficacy in treatment. It should also be tested using the longer version of the IAT (Young, 1998; Widyanto and McMurran, 2004) as a more tested measure in the literature. We used the shorter version given the length of the assessment tool we used for the whole model but if replicating this work with a clinical sample, it would be suggested to use the IAT along with additional measures of IA, such as the Assessment of Internet and Computer game Addiction as scale (AICA-S) or clinical interview (AICA-C) developed and validated with clinical groups by (Wölfling et al., 2010, 2012). Furthermore, we developed and tested the Internet use expectancies questionnaire for the purposes of this study. While we were methodologically conservative and careful in the development of the scale, this measure should be evaluated on additional populations for validity and the questionnaire needs further empirical testing in future studies. Additional and more detailed scales and interviews should also be applied to clinical samples, since most of the facets assessed in our study were measured using short questionnaires with a restrictive number of items, due to practical reasons (time limitation in the context of online surveys). A further potential problem is that of common method variance (Podsakoff et al., 2003). Unfortunately, no clear marker variable, which should theoretically be unrelated to all other variables, has been included in the study for practical reasons (the survey took almost 25 min, which is a critical threshold for online surveys). Although we cannot exclude the effect of common method variance on the results, we argue that this effect unlikely account for the whole data structure reported. When inspecting the bivariate correlations (**Table 3**) one can see that some of those are very low (e.g., *r* = –0.08, *r* = –0.09, *r* = 0.12 etc.). We think that these low correlations give some tender hints for the assumption that common method variance does not affect the main analyses dramatically. Nonetheless, the model should be tested with a systematic multi-trait-multi-method approach (Campbell and Fiske, 1959) in future studies.

The current study focuses on GIA, which means that the model on SIA, as described by Brand et al. (2014), still needs to be tested empirically. Different forms of SIA (e.g., gaming, online porn, or Internet gambling) should be tested to see if coping skills and Internet use expectancies play a similar role in development of the problem. It is also still a debate if the concept of GIA is principally adequate for covering the problematic behavior in patients. We found evidence for the link between self-reported problems related to an unspecific use of several different Internet applications and the variables suggested in the model. The concept of GIA was operationalized by the s-IAT instruction and item formulations, but also by the fact that more than 97% of the participants reported to regularly use three or more different Internet applications, such as communication, gaming, gambling, cybersex, shopping, or information seeking. From a clinical perspective, it is nevertheless a topic of debate if GIA can be a reason for seeking treatment or if treatment-seeking patients basically suffer from a loss of control over the use of one certain application, only. We suggest to consider this point in clinical research by systematically investigate the critical behavior in the context of the Internet use and analyze how frequent the uncontrolled and addictive use of more than one Internet application is in clinical samples. In addition, not all components proposed in the theoretical model on GIA could be included in this study. For example, additional personality traits or other psychopathological disorders may be included in future studies.

## CONCLUSION

The main hypotheses of the model on GIA are supported by empirical data. Person’s core characteristics are related to symptoms of GIA, but these effects are mediated by person’s specific cognitions, in particular coping style and Internet use expectancies. These cognitions should be addressed in the treatment of an addictive use of the Internet.

## AUTHOR CONTRIBUTIONS

Matthias Brand wrote the first draft of the paper, supervised the data collection, and analyzed and interpreted the data. Christian Laier contributed particularly to the conceptualization of the empirical study and data collection, and revised the manuscript. Kimberly S. Young edited the draft, revised it critically, and contributed intellectually and practically to the manuscript. All authors finally approved the manuscript. All authors are accountable for all aspects of the work.

## Conflict of Interest Statement

The authors declare that the research was conducted in the absence of any commercial or financial relationships that could be construed as a potential conflict of interest.
